# Supplementary material for: Enarodustat suppresses thymic stromal lymphopoietin expression via hypoxia-inducible factor-mediated c-Jun N-terminal kinases dephosphorylation
Source: PLoS One. 2026 Jan 23;21(1):e0341552. doi: 10.1371/journal.pone.0341552 (PMC12829785; doi:10.1371/journal.pone.0341552)

# Original western blot data

Fig. S1. Original western blot data related to Fig. 2.

A

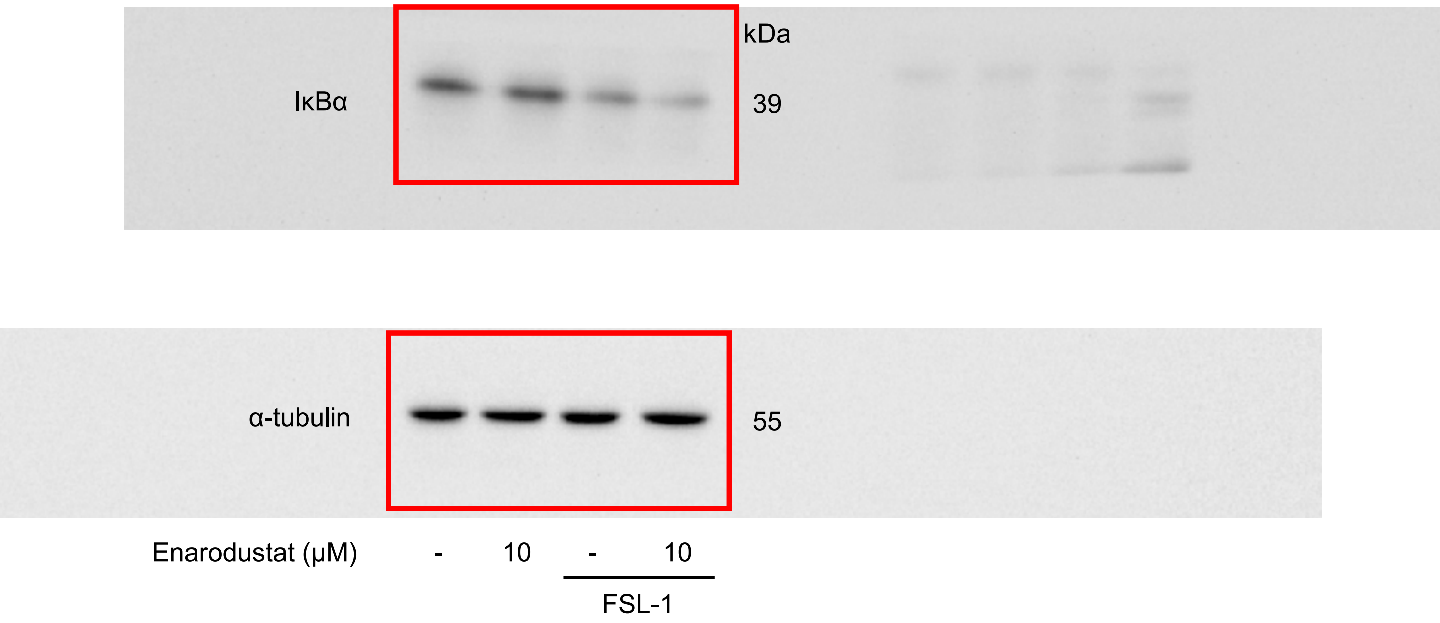


B

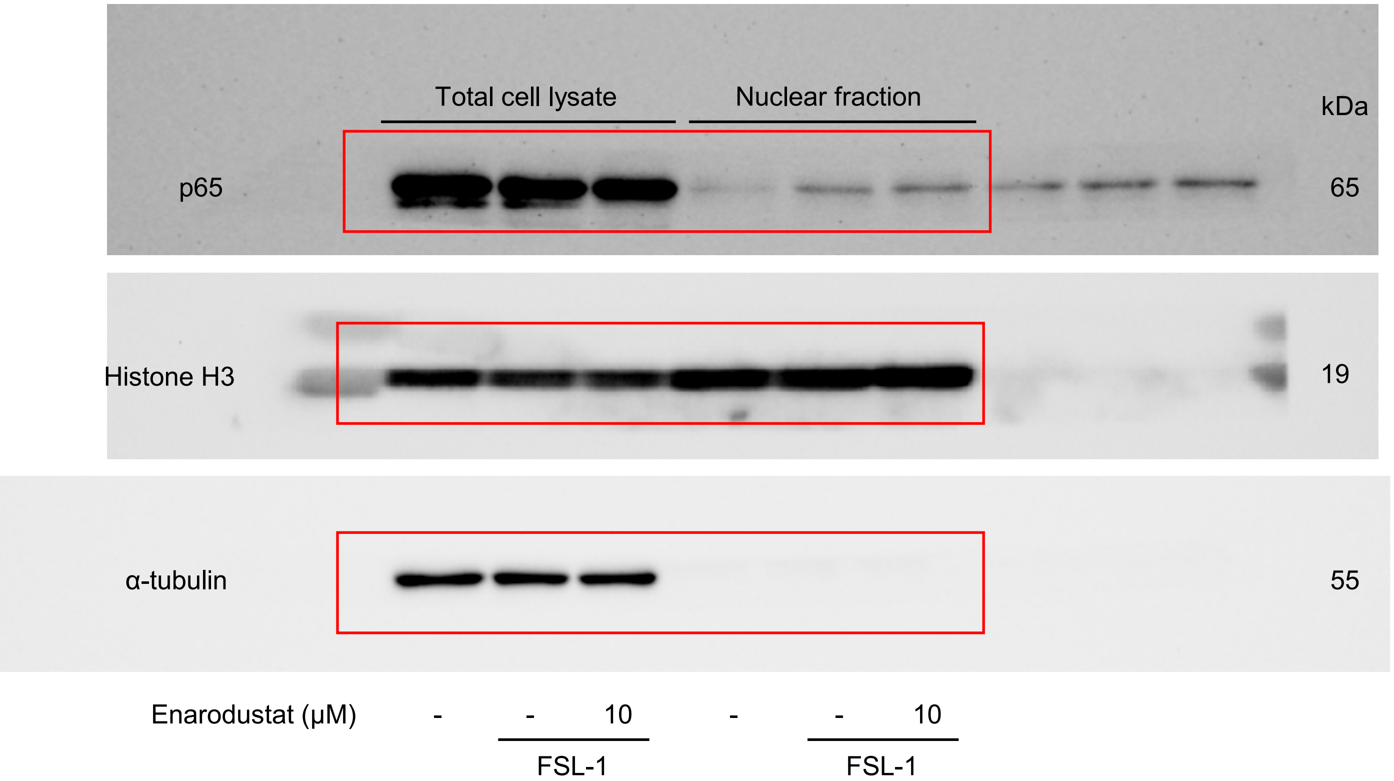

Fig. S2. Original western blot data related to Fig. 3.

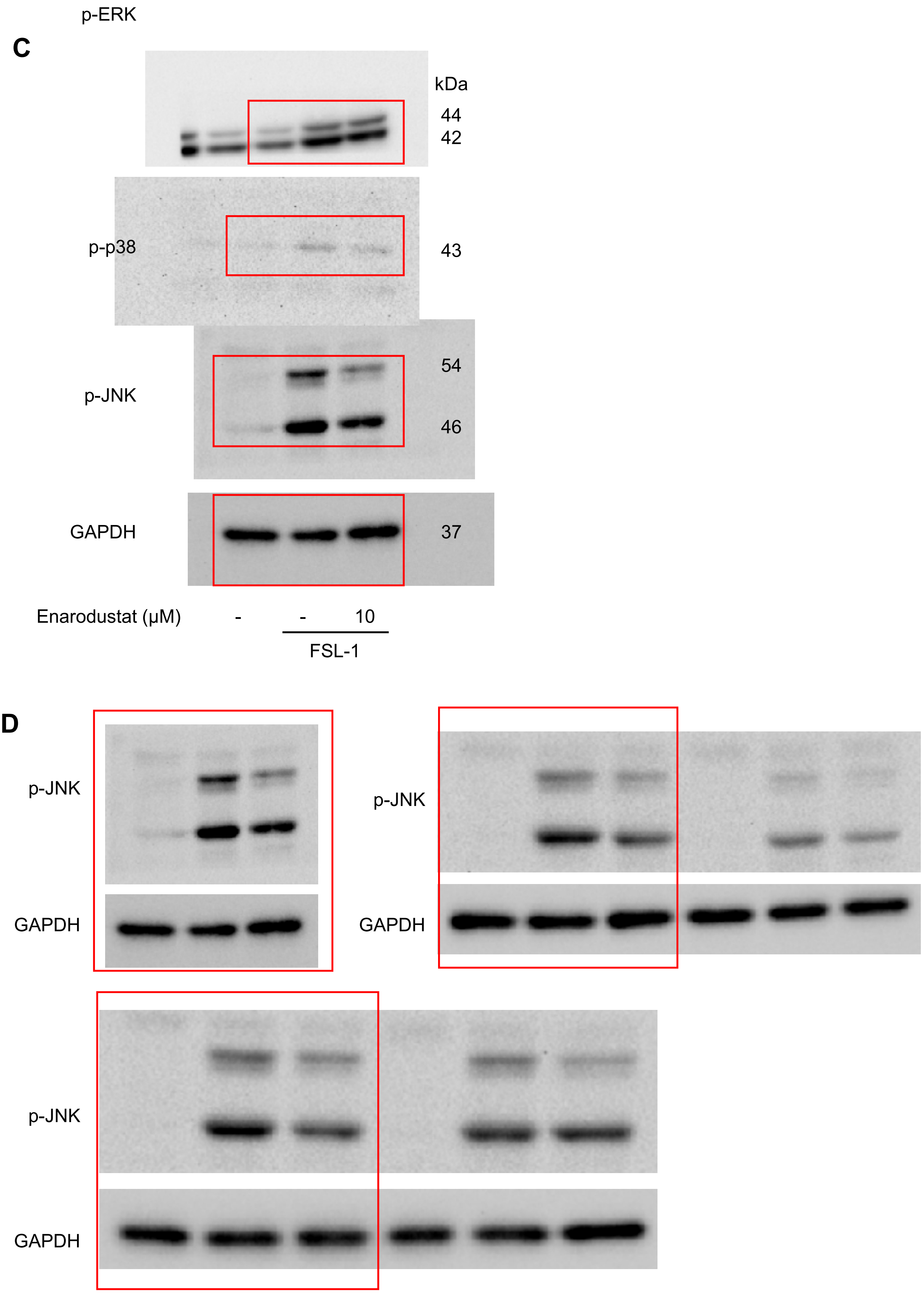

Fig. S3. Original western blot data related to Fig. 4.

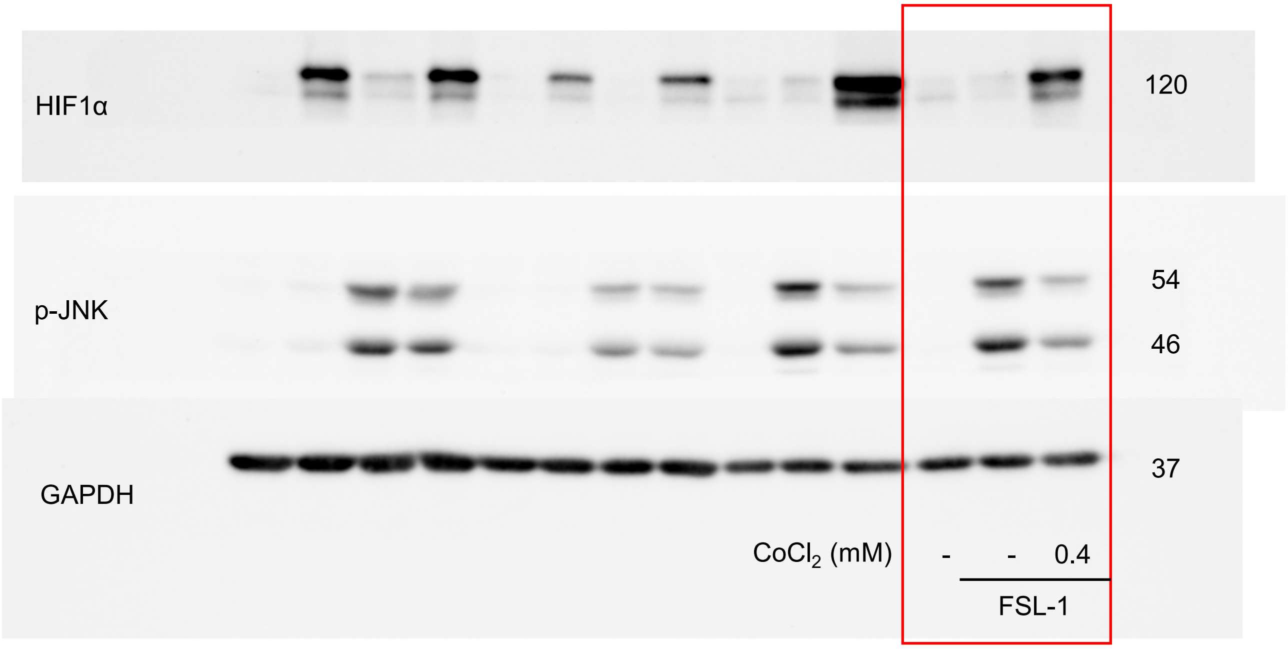

Fig. S4. Original western blot data related to Fig. 5.

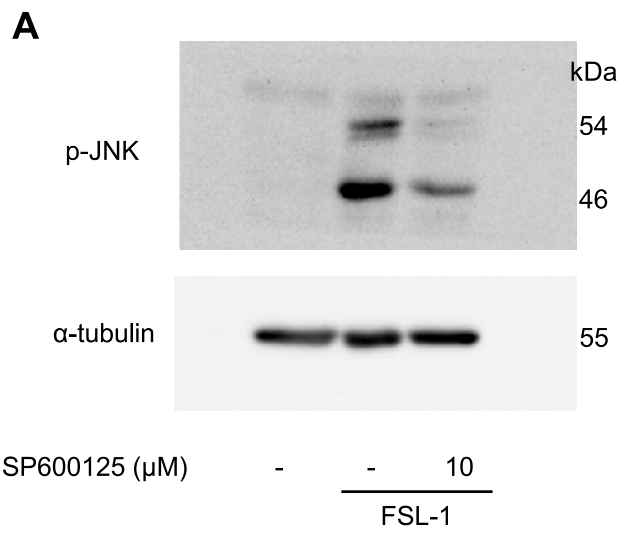


Fig. S5. Original western blot data related to Fig. 7.

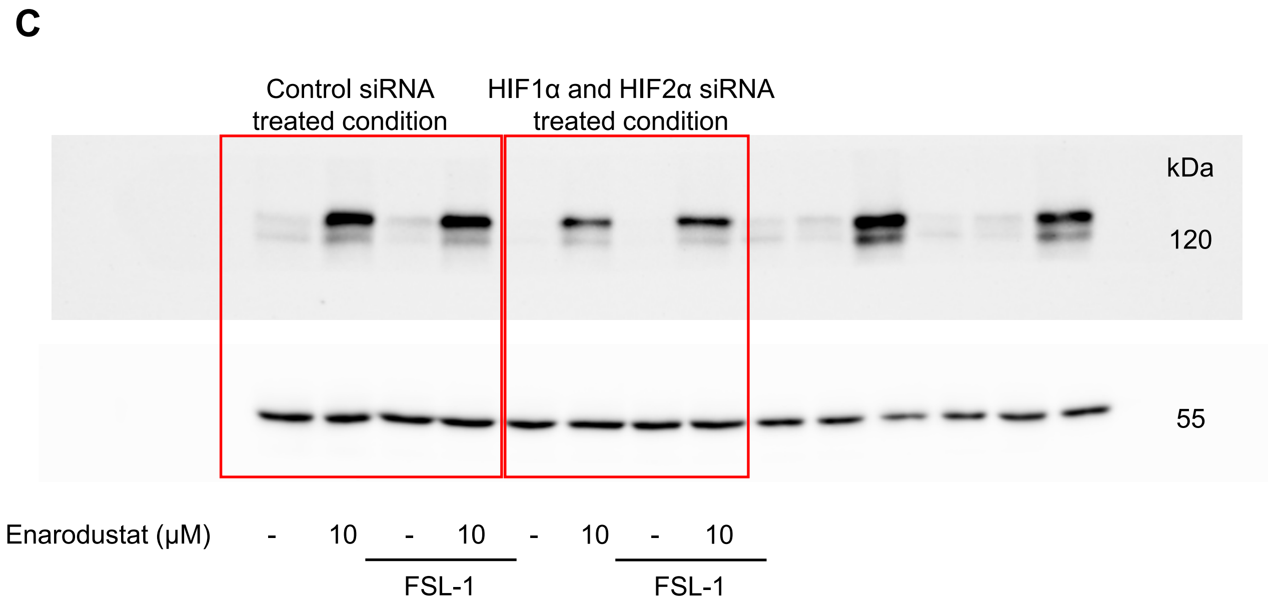

Fig. S6. Original western blot data related to Fig. 8.

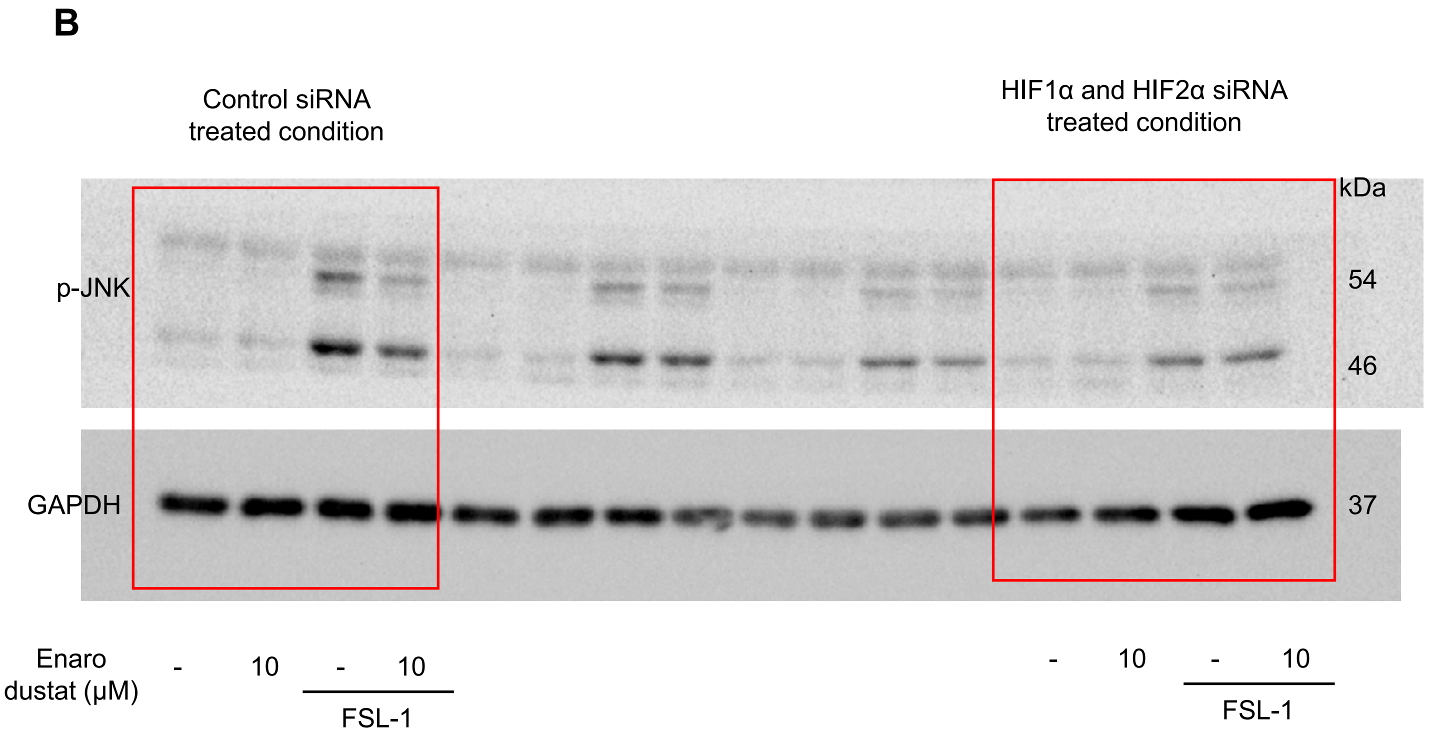

Supplement: S1 raw images — (PDF) [file pone.0341552.s001.pdf]
